# The Hierarchy of Coupled Sleep Oscillations Reverses with Aging in Humans

**DOI:** 10.1523/JNEUROSCI.0586-23.2023

**Published:** 2023-09-06

**Authors:** Marc Alain Züst, Christian Mikutta, Ximena Omlin, Tatjana DeStefani, Marina Wunderlin, Céline Jacqueline Zeller, Kristoffer Daniel Fehér, Elisabeth Hertenstein, Carlotta L. Schneider, Charlotte Elisabeth Teunissen, Leila Tarokh, Stefan Klöppel, Bernd Feige, Dieter Riemann, Christoph Nissen

**Affiliations:** ^1^University Hospital of Old Age Psychiatry and Psychotherapy, University of Bern, 3000 Bern 60, Switzerland; ^2^University Hospital of Psychiatry and Psychotherapy, University of Bern, 3000 Bern 60, Switzerland; ^3^Private Clinic Meiringen, 3860 Meiringen, Switzerland; ^4^Department of Physiology, Anatomy and Genetics, University of Oxford, Oxford OX1 3PT, United Kingdom; ^5^Division of Psychiatric Specialties, Geneva University Hospitals (HUG), 1201 Geneva, Switzerland; ^6^Neurochemistry Laboratory, Department of Clinical Chemistry, Amsterdam Neuroscience, Neurodegeneration, Amsterdam UMC, Vrije Universiteit Amsterdam, 1081 HV, Amsterdam, The Netherlands; ^7^University Hospital of Child and Adolescent Psychiatry and Psychotherapy, University of Bern, 3000 Bern 60, Switzerland; ^8^Department of Psychiatry and Psychotherapy, University of Freiburg Medical Center, 79104 Freiburg, Germany

**Keywords:** aging, human life span, neurodegeneration, phase–amplitude coupling, sleep spindles, slow-wave sleep

## Abstract

A well orchestrated coupling hierarchy of slow waves and spindles during slow-wave sleep supports memory consolidation. In old age, the duration of slow-wave sleep and the number of coupling events decrease. The coupling hierarchy deteriorates, predicting memory loss and brain atrophy. Here, we investigate the dynamics of this physiological change in slow wave–spindle coupling in a frontocentral electroencephalography position in a large sample (*N* = 340; 237 females, 103 males) spanning most of the human life span (age range, 15–83 years). We find that, instead of changing abruptly, spindles gradually shift from being driven by slow waves to driving slow waves with age, reversing the coupling hierarchy typically seen in younger brains. Reversal was stronger the lower the slow-wave frequency, and starts around midlife (age range, ∼40–48 years), with an established reversed hierarchy between 56 and 83 years of age. Notably, coupling strength remains unaffected by age. In older adults, deteriorating slow wave–spindle coupling, measured using the phase slope index (PSI) and the number of coupling events, is associated with blood plasma glial fibrillary acidic protein levels, a marker for astrocyte activation. Data-driven models suggest that decreased sleep time and higher age lead to fewer coupling events, paralleled by increased astrocyte activation. Counterintuitively, astrocyte activation is associated with a backshift of the coupling hierarchy (PSI) toward a “younger” status along with increased coupling occurrence and strength, potentially suggesting compensatory processes. As the changes in coupling hierarchy occur gradually starting at midlife, we suggest there exists a sizable window of opportunity for early interventions to counteract undesirable trajectories associated with neurodegeneration.

**SIGNIFICANCE STATEMENT** Evidence accumulates that sleep disturbances and cognitive decline are bidirectionally and causally linked, forming a vicious cycle. Improving sleep quality could break this cycle. One marker for sleep quality is a clear hierarchical structure of sleep oscillations. Previous studies showed that sleep oscillations decouple in old age. Here, we show that, rather, the hierarchical structure gradually shifts across the human life span and reverses in old age, while coupling strength remains unchanged. This shift is associated with markers for astrocyte activation in old age. The shifting hierarchy resembles brain maturation, plateau, and wear processes. This study furthers our comprehension of this important neurophysiological process and its dynamic evolution across the human life span.

## Introduction

Sleep is of central importance for the brain, promoting vital functions like memory consolidation, synaptic renormalization, and clearance of metabolic waste-products like amyloid β (Aβ), a hallmark for Alzheimer's disease (Rasch and Born, 2013; Xie et al., 2013; Mander et al., 2016; Tononi and Cirelli, 2020). Coupled oscillations, especially during slow-wave sleep (SWS), have been identified as a cornerstone of the function of sleep for the brain. Neocortical slow waves (SWs; <1.25 Hz), thalamocortical spindles (12–16 Hz) and hippocampal sharp-wave ripples (80–100 Hz) are hierarchically orchestrated to allow for optimized, synchronized information processing that enables memory consolidation (Rasch and Born, 2013; Staresina et al., 2015). For optimal functionality, the layers of this hierarchy are organized in a relationship of phase-amplitude coupling, where the faster spindles are nested into the depolarizing up-phase of the slower SWs. This allows for synchronized, widespread communication during periods of high responsiveness and therefore efficient top-down control of processes like memory consolidation (Tort et al., 2010; Rasch and Born, 2013; Helfrich et al., 2018; Mikutta et al., 2019; Bastian et al., 2022).

With age, sleep quality and quantity declines, leading to a loss of SWS (Mander et al., 2017). This loss inevitably entails less opportunity for sleep's important functions. While part of normal aging (Carrier et al., 2011; Hertenstein et al., 2018), this loss is more severe in neurodegenerative disorders, like Alzheimer's disease (Rauchs et al., 2008; Westerberg et al., 2012; Zhang et al., 2022). As neurodegeneration progresses, sleep quality declines, which in turn robs the brain of crucial recuperative functions, worsening neurodegeneration (Mander et al., 2016). With lacking SWS, Aβ is not cleared from the brain as effectively, and the residual Aβ in turn disrupts sleep (Kang et al., 2009; Roh et al., 2012; Mander et al., 2015; Varga et al., 2016; Ju et al., 2017; Fultz et al., 2019; Winer et al., 2019, 2020; Eide et al., 2021), leading to a vicious cycle (Mander et al., 2016; Wunderlin et al., 2020; Zeller et al., 2023).

The orchestrated coupling of spindles and SWs follows along with these age-related sleep changes. Recent studies posit that spindles become uncoupled from SWs in the aging brain, and this change is associated with degrading memory and medial frontal brain atrophy (Helfrich et al., 2018; Muehlroth et al., 2019). In younger individuals, SW drive spindles, signifying that SWs inhabit a higher position in the hierarchy of coupled oscillations. In older individuals, however, this clear cross-frequency directionality deteriorates (Helfrich et al., 2018).

Importantly, it is known that older individuals with higher structural brain integrity in areas like the medial prefrontal cortex and hippocampus exhibit a SW–spindle coupling physiology reminiscent of a younger brain (Muehlroth et al., 2019). Moreover, enhancing SW–spindle coupling using transcranial electric stimulation has been shown to improve postsleep declarative memory retrieval in older adults with mild cognitive impairment (Ladenbauer et al., 2017), suggesting that the unfavorable age-associated deterioration of SW–spindle coupling can potentially be compensated to prevent cognitive decline.

While currently available research paints quite a stark contrast between younger and older adults, it is not clear how and when these changes emerge. Are changes in SW–spindle coupling gradually appearing across the adult human life span, or suddenly at a specific age? At what age does the process become apparent? Here, we address these open questions by examining SW–spindle coupling in an extensive sample (*N* = 340) spanning a large portion of the human life span (age range, 15–83 years). Instead of focusing on group differences between younger and older individuals including all associated cross-generational inhomogeneity, we investigate SW–spindle coupling as a continuum across the human life span.

When aiming to prevent cognitive decline, the early detection of unfavorable trajectories is key. Recently, blood-based biomarker assessments have become an affordable, minimally invasive approach for the early prediction of cognitive decline (Verberk et al., 2020; Thijssen et al., 2021; Beyer et al., 2022). The most promising prognostic blood-based biomarkers currently discussed are Aβ42/40 ratio and glial fibrillary acidic protein (GFAP) levels. A lower blood Aβ42/40 ratio is thought to be a marker for impaired clearance of Aβ from the brain. Increased levels of GFAP is a marker for astrocyte activation, with a potential role in neuroinflammation because of neuronal damage or degeneration (Verberk et al., 2020; Thijssen et al., 2021). Experimentally induced sleep deprivation is linked with astrocyte activation and neuroinflammation, as indicated by increased cytokine and GFAP levels in rodents (Manchanda et al., 2018; Xiao et al., 2022). High GFAP levels are associated with a steeper rate of decline in memory, executive functioning, and attention, and had a high prognostic value for incident dementia in humans (Verberk et al., 2021). Using a combination of amyloid misfolding status and GFAP levels, the incidence of Alzheimer's disease diagnosis could be accurately predicted 17 years in advance with a receiver-operating characteristic area under the curve of 0.83 (Beyer et al., 2022), paving the way for minimally invasive early detection of cognitive decline.

In addition to investigating the dynamics of the shift in SW–spindle coupling across the human life span, we examine whether changes in the hierarchical coupling structure of brain oscillations during slow-wave sleep are reflective of neuronal degradation as measured by blood-based biomarkers. For this purpose, we analyze associations of SW–spindle coupling with readily accessible blood-based biomarkers for dementia and astrocyte activation (Aβ42/40 ratios and GFAP levels) in a subgroup of older individuals.

A continuous investigation of brain physiology from adolescence to senescence allows for a deeper understanding of the dynamic processes the brain undergoes throughout our lifetime. It can put individual neurophysiological characteristics into context, allow us to better identify pathologic trajectories, and separate pathologic from healthy trajectories. This knowledge can accelerate the development of tailored treatment and prevention methods for cognitive decline, especially as more early warning signs are identified every year.

## Materials and Methods

### Sample.

The total sample consisted of 340 whole-night baseline sleep recordings of healthy participants (237 females, 103 males; age range, 15–83 years; mean ± SD age, 43.4 ± 17.8 years; Table 1) participating in various studies at the Department of Psychiatry and Psychotherapy, University of Freiburg Medical Center (UFMC) between 2008 and 2018 and University Hospital for Old Age Psychiatry and Psychotherapy Bern (UPD) between 2019 and 2021. Of the total sample, 310 participants were measured at UFMC (213 females; age range, 15–83 years; mean ± SD age, 40.9 ± 16.5 years) and 30 participants were measured at UPD (24 females; age range, 61–80 years; mean ± SD age, 69.5 ± 4.3 years). All participants underwent extensive screening procedures to confirm suitability as healthy study participants, which was the first inclusion criterion. The second inclusion criterion was the availability of polysomnography (PSG) recordings of an entire night under baseline measurement conditions after an adaptation night (i.e., natural sleep with no intervention). Exclusion criteria were current or recent (over the last 6 months) psychiatric or physical illness, especially if impacting sleep (e.g., insomnia, hypersomnia, sleep apnea syndrome, or restless legs syndrome), irregular sleep patterns, substance abuse, use of prescription medication acting on the CNS, and pregnancy. Studies were conducted in accordance with the Declaration of Helsinki as approved by local ethics committees. All individuals (and their parents if underage) gave written informed consent.

**Table 1.** Sleep parameters

	ALL (*N* = 340)	Q1 (age, 15–27 years; *N* = 84)	Q2 (age, 28–46 years; *N* = 85)	Q3 (age, 47–56 years; *N* = 85)	Q4 (age, 57–83 years; *N* = 86)	*R* ^2^_age_
SPT, h	7.8 ± 0.8	8.4 ± 0.5	7.7 ± 0.3	7.6 ± 0.8	7.6 ± 1.0	0.14↓
TST, h	7.0 ± 1.0	7.9 ± 0.6	7.0 ± 0.6	6.8 ± 0.8	6.2 ± 1.1	0.43↓
SL, h	0.9 ± 0.8	0.6 ± 0.7	0.9 ± 0.8	1.0 ± 0.8	0.9 ± 1.0	<0.01
Wake %	11.1 ± 8.3	5.2 ± 3.9	9.7 ± 5.7	11.1 ± 6.1	18.4 ± 10.0	0.37↑
Stage N1, %	9.7 ± 6.7	5.6 ± 2.9	7.8 ± 3.7	10.5 ± 6.6	14.9 ± 8.0	0.31↑
Stage N2, %	50.1 ± 9.5	47.4 ± 7.5	53.8 ± 6.7	53.3 ± 7.9	45.8 ± 12.3	<0.01
Stage N3, %	10.5 ± 9.5	21.3 ± 8.8	8.5 ± 7.1	6.3 ± 6.4	5.8 ± 6.2	0.38↓
Stage R, %	18.6 ± 5.0	20.5 ± 4.3	20.2 ± 4.0	18.8 ± 4.9	15.1 ± 4.9	0.18↓
Sleep efficiency, %	88.9 ± 8.3	94.8 ± 3.9	90.3 ± 5.7	88.9 ± 6.1	81.6 ± 10.0	0.37↓
SD	9.3 ± 1.6	10.4 ± 0.8	9.8 ± 1.2	9.2 ± 1.4	7.8 ± 1.4	0.41↓
SW amp	60.6 ± 25.6	84.9 ± 22.1	58.1 ± 25.6	47.8 ± 15.8	51.9 ± 20.1	0.25↓
NCE, ×10^3^	1.8 ± 0.6	2.3 ± 0.4	1.8 ± 0.4	1.7 ± 0.5	1.2 ± 0.5	0.48↓

Sleep stages (N1 to N33, R) and intermittent wakefulness (Wake) as a percentage of SPT, sleep efficiency as percentage of sleep during bedtime, spindle density during N2/N3 in spindle events per minute, SW amp in µV, number of coupling events (NCE) in thousands; all mean ± SD. The first data column represents the whole sample (*N* = 340), the columns Q1 to Q4 represent age quartiles Q1 to Q4 of the whole sample. The last column indicates age trends and explained variance by age (*R*^2^_age_, Pearson's determination coefficient). ↓, Trending down, *p* < 0.001; ↑, trending up, *p* < 0.001.

### Procedures.

All participants completed 1 night of PSG. At UFMC, sleep was recorded on a 24-channel EEG PSG device with a sampling rate of 200 or 256 Hz. Recorded EEG channels included C3, C4, Fz, Fpz, and Oz. During recording, channels C3 and C4 were referenced against contralateral mastoids, and the other channels were referenced against pooled mastoids or Cz. More information about UFMC data, infrastructure, and standard procedures have been reported previously (Hertenstein et al., 2018). At UPD, sleep was recorded using a high-density EEG system (128-channel MicroCel Geodesic Sensor Net, 16-channel Physio16 input box, 400 Series Geodesic EEG System) by Magstim EGI, with a sampling rate of 500 Hz, referenced against Cz. Polysomnographic scoring of sleep stages was performed according to the criteria of the American Academy of Sleep Medicine (Iber et al., 2007) by experienced somnologists for all 340 datasets.

For 28 of the 30 participants in the UPD sample (22 females; age range, 61–80 years; mean ± SD age, 69.5 ± 3.9 years), blood samples were taken in the morning (∼1 h after waking) and immediately centrifuged and stored at −80°C. The resulting plasma samples were analyzed in the Neurochemistry Laboratory, Amsterdam University Medical Center (Amsterdam, The Netherlands). Plasma Aβ 1–42 and 1–40, as well as GFAP levels were quantified using single molecule array immunoassays (IA-N4PE; Thijssen et al., 2021), and Aβ42/40 ratios were calculated. All measurements were above the limits of detection and the functional lower limits of quantification as per the manufacturer specifications. High GFAP levels and low Aβ42/40 ratios constitute risk factors for neurodegenerative disease and are strongly associated with amyloid positivity as assessed with positron emission tomography (Graff-Radford et al., 2007; Verberk et al., 2020, 2021).

### Sleep parameters.

We determined the following sleep parameters individually, then averaged for the whole sample (*N* = 340) as well as for age quartiles: sleep period time (SPT), defined as the time from sleep onset to the final awakening (Hertenstein et al., 2018); total sleep time (TST; i.e., SPT minus intermittent wakefulness); sleep (onset) latency (SL; i.e., the time until first occurrence of non-rapid eye movement sleep stage 1); sleep efficiency as a percentage of sleep during bedtime; spindle density (measured as spindle events per minute of N2/N3 sleep); SW amplitude (SW amp; in µV; negative-to-positive peak of detected SW events), as well as the standard American Academy of Sleep Medicine sleep architectural stages [i.e., wakefulness, non-rapid eye movement (non-REM) sleep stage 1 (N1) through N3, and REM sleep]. SPT, TST, and SL are measured in hours, and sleep architectural stages are measured as the percentage of SPT. For all sleep parameters, we tested the association with age using Pearson's determination coefficients. A more in-depth evaluation of the sleep parameters of UFMC data have been reported previously (Hertenstein et al., 2018).

### EEG processing.

EEG processing, as well as calculation and statistical analysis of SW–spindle coupling was achieved in MATLAB R2019a (MathWorks) using EEGLAB (Delorme and Makeig, 2004), the CircStat toolbox (Berens, 2009), the FieldTrip toolbox (Oostenveld et al., 2011), and the phase-amplitude coupling analysis framework by Jiang et al. (2015). For the UFMC dataset, 30 s segments of data containing artifacts were manually labeled and excluded from analysis. For the UPD dataset, EEG data were preprocessed using the PREP pipeline for EEGLAB (Bigdely-Shamlo et al., 2015) and the automatic artifact rejection pipeline as implemented in the FieldTrip toolbox (Oostenveld et al., 2011). All analyses were conducted on artifact-free N2 or N3 sleep data on channel Fz, referenced against pooled mastoids, resampled to 200 Hz if necessary.

### Slow-wave, spindle, and coupling event classification.

SW and spindle events were detected using previously established methods (Mölle et al., 2009; Staresina et al., 2015; Helfrich et al., 2018). For slow oscillations, we filtered data between 0.16 and 1.25 Hz and marked zero crossings. SW events were then defined as negative peaks between two consecutive positive-to-negative zero crossings based on duration (0.8–2 s) and amplitude (individual 75th percentile) criteria (Mölle et al., 2009; Helfrich et al., 2018). For sleep spindles, we filtered data between 12 and 16 Hz and extracted the amplitude of the Hilbert transform. Spindle events were defined as peaks of the smoothed (200 ms moving average) Hilbert amplitude curve in regions that exceeded the individual 75th amplitude percentile for 0.5–3 s (Staresina et al., 2015). Spindle events that were within 2.5 s of an SW event were marked as SW-coupled spindles and constitute coupling events. We then extracted the coupling phases [i.e., the instantaneous SW phase angles of SW-coupled spindles using the angle of the Hilbert transform in SW-filtered (0.16–2 Hz) data]. To counteract reduced SW power with age, we *z*-standardized data within participants before analyses of SW–spindle coupling.

### Quantifying slow wave–spindle coupling.

The number of coupling events yields a first measure of SW–spindle coupling and can vary with quantity (i.e., the time spent asleep) and/or quality (i.e., the exact electrophysiological synchronization of SWs and spindles) of SWS. In addition to the number of coupling events, we calculated the following three principal SWS-quantity-independent measures of SW–spindle coupling.

1. The resultant vector (rvec) angle, or mean circular direction (CircStat::circ_mean) of coupling phases yields a measure of the preferred coupling phase of spindles within SWs. An rvec angle of 0° is equivalent to the positive peak, ±180° to the negative peak, negative values up to –90° are before a positive peak, and positive values up to 90° are after a positive peak. As rvec angle is a circular measure, its utility is limited to circular statistics, and it cannot be included in linear models.
2. The modulation index MI; Tort et al., 2010; Jiang et al., 2015) as a measure of cross-frequency coupling was calculated between the phase of a lower frequency (SWs, 0.39–1.95 Hz in 0.39 Hz steps) and the amplitude of a higher frequency (spindles, 12–16 Hz in 1 Hz steps). The MI is a measure of circular spread and indicates how far an empirical distribution deviates from uniformity using the Kullback–Leibler divergence. The higher the MI, the more closely all coupling phases are grouped around the preferred phase (i.e., the stronger the coupling).
3. The phase slope index (PSI; Jiang et al., 2015) as a measure of cross-frequency directionality was calculated between the phase of a lower frequency (SWs, 0.5–2 Hz in 0.5 Hz steps) and the amplitude of a higher frequency (spindles, 12–16 Hz in 1 Hz steps). The PSI robustly measures the consistency of phase lag or lead between the two frequencies, and a value significantly different from 0 is suggestive of causal influence of the leading over the lagging frequency. A positive PSI indicates that SWs drive spindles, and a negative PSI indicates that spindles drive SWs. The PSI can be used instead of rvec angle in linear models.

MI and PSI were calculated on 5 s data segments centered on the negative peak of detected slow waves. We defined a sliding window of 2 s length with 1 s steps, using five cycles to estimate frequency power. We then averaged the resulting MI and PSI values for all possible frequency subband pairs to yield a single estimate for MI and PSI between the SW and spindle bands per subject.

As the number of coupling events diminishes with age (*R*^2^ = 0.48, *p* < 0.001), lower numbers of coupling events might bias the estimation of SW–spindle coupling and its association with age. To counteract this, we implemented a per-subject bootstrapping procedure where we repeated the calculation of rvec angles and MI with *q* randomly selected coupling events, where *q* equals the smallest number of coupling events across all participants (*q* = 120 in a subject 77 years of age). This random draw was repeated for 1000 iterations per subject (or, if not possible, for the maximum number of unique draws), and an average coupling measure was then calculated from the mean of the bootstrapping distribution. We used a leave-one-out jackknifing procedure to test stability of the estimation of the PSI. If estimation of the PSI exhibited low stability (|*z*(jackknifing error)| > 2), the subject was excluded from PSI analyses, which was the case in 9 of 340 participants. These unstable estimates contained three outliers (|*z*(PSI)| > 3), and no outliers remained after the exclusion of unstable estimates.

### Testing for the association of slow wave–spindle coupling and age.

We tested for associations of measures of SW–spindle coupling (number of coupling events, rvec angle, MI, and PSI) with age. For linear coupling measures (number of coupling events, MI, and PSI), we calculated the Pearson correlation coefficient with age. For the preferred coupling phase (rvec angle), we used CircStat::circ_corrcl for a circular–linear correlation between rvec angle and age. Importantly, an rvec angle can technically be calculated even in almost uniformly distributed data, but would not produce a sensible estimate of preferred phase in that case. Therefore, we repeated the circular–linear correlation between rvec angle and age, as well as the linear correlation between PSI and age, in a subset of individuals exhibiting high coupling strength as measured by MI to minimize bias because of invalid estimations of preferred phase. As MI was right skewed, we defined high MI as *z*(ln(MI)) > 0, which was the case in 177 participants. This logarithmic transformation normalizes the distribution of MI and allows *z*-transformed values >0 to represent the upper half of MI data. For effect sizes, we calculated the explained variance through determination coefficients (*R*^2^).

For significant associations of SW–spindle coupling with age (rvec angle and PSI), we further subdivided the sample into age quartiles and tested the quartiles separately against zero. Preferred coupling phase (rvec angle) was tested against zero using a one-sample test for mean angle (CircStat::circ_mtest). Zero was chosen as test value because it marks the highest point on a positive SW peak, thus allowing testing if spindles prefer to nest significantly before or after a SW peak. PSI was tested against zero using one-sample *t* tests. Zero was chosen as test value because it marks the reversal-point of cross-frequency directionality (i.e., a reversal of which frequency drives the other). We additionally calculated the *x*-zero-crossing of the best-fit models of the association between SW–spindle coupling measures and age to estimate the age at which a reversal happens. For MI, we tested age quartiles against each other in an ANOVA to test for potential nonlinear shifts in coupling strength between age quartiles.

To account for potential sources of bias, we calculated a linear regression of age on PSI (Matlab::lmfit) and let a data-driven stepwise procedure (Matlab::step) optimize this model by testing change in model fit by the inclusion and exclusion of terms. Each step, the term yielding the highest gain in *R*^2^ is added, provided *R*^2^ would increase by at least 0.1. Gender, age, linear coupling measures, SW up-phase and down-phase duration, number of coupling events, sleep parameters (TST, SL, and SPT in hours; stages N1 to N3, and REM; as well as intermittent wakefulness in the percentage of SPT, SD, sleep efficiency, and SW amp), and interactions of existing terms may be added as factors if not already present. If no term can be added this way, the term resulting in the least loss of *R*^2^ is removed provided that *R*^2^ would decrease by no more than 0.05. If neither threshold is met through further changes in the model, the procedure ends. Final models were *F*-tested against intercept-only models. To prevent overfitting, model *R*^2^ was adjusted for the number of included terms. To explicitly test for an influence of up-phase and down-phase duration on coupling, an additional model was calculated to include up-phase and down-phase duration a priori. To explicitly test for gender differences, an additional model was calculated to include gender a priori. For these models, the Δ*R*^2^ threshold for excluding terms was set more liberally at 0.02 to allow control for gender and SW duration effects even if they are small. All model optimizations finished within four steps.

### Blood biomarker analysis.

For 28 participants in the UPD sample, blood-based biomarkers were analyzed for associations with SW–spindle coupling while controlling for potential confounders such as gender, age, and sleep parameters. Initially, a priori baseline models were defined explaining blood biomarkers (Aβ42/40 ratios and GFAP levels) by linear coupling measures (number of coupling events, MI, and PSI) and age, and explaining linear coupling measures by blood biomarkers and age. Stepwise optimization allowed for the inclusion and exclusion of gender, age, blood biomarkers, linear coupling measures, sleep parameters, and interactions as described above (subsection Testing for association of slow wave–spindle coupling and age). All model optimizations finished within three steps.

### Data availability.

Data will be deposited on an open repository (e.g., https://boris-portal.unibe.ch/) on article acceptance.

## Results

### Trends for sleep parameters across the human life span replicate earlier findings

Consistent with earlier studies (Carrier et al., 2011; Hertenstein et al., 2018), the structure of sleep changes with age (Table 1). We found significant decreases in SPT (*R*^2^ = 0.14), TST (*R*^2^ = 0.43), proportional non-REM sleep stages N3 (*R*^2^ = 0.38) and REM sleep (*R*^2^ = 0.18) sleep, as well as spindle density (*R*^2^ = 0.41), SW amplitude (*R*^2^ = 0.25), and the number of coupling events (*R*^2^ = 0.48) with age (*p* < 0.001). Conversely, N1 sleep (*R*^2^ = 0.31) and periods of intermittent wakefulness (*R*^2^ = 0.37) were increased (*p* < 0.001) with age. SL and stage N2 did not change with age (*R*^2^ < 0.01, n.s.).

### Spindle density, age, and total sleep time determine number of slow wave–spindle coupling events

With age the number of coupling events is strongly reduced (*R*^2^ = 0.48, *p* < 0.001; Table 1). A stepwise optimized linear regression model (*F*_(1,338)_ = 1910, *R*^2^_adj_ = 0.85, *p* < 0.001) indicated that the number of coupling events is best explained by spindle density (*t* = 43.69, *p* < 0.001), an association so strong that no other factors were being considered. If spindle density was removed from the pool of available regressors, an optimized model (*F*_(2,337)_ = 282, *R*^2^_adj_ = 0.62, *p* < 0.001) indicated that the number of coupling events is best explained by age (*t* = −8.24, *p* < 0.001) and TST (*t* = 11.51, *p* < 0.001). It is therefore difficult to isolate the effect of age on SW–spindle coupling measures (rvec angle, MI, and PSI) from reduced numbers of coupling events because of reduced spindle density. To counteract this, we implemented bootstrapping and jackknifing procedures (see Materials and Methods, subsection Quantifying slow wave–spindle coupling) to test the robustness of age effects on SW–spindle coupling measures against variance in the number of coupling events. This allows the evaluation of age-related effects on SW–spindle coupling while the number of coupling events is held constant. Notably, all results regarding age effects in rvec angle, MI, and PSI are unchanged whether these procedures are implemented or not.

### Spindles shift from lagging to leading slow waves without loss of coupling strength with age

**Figure 1.**
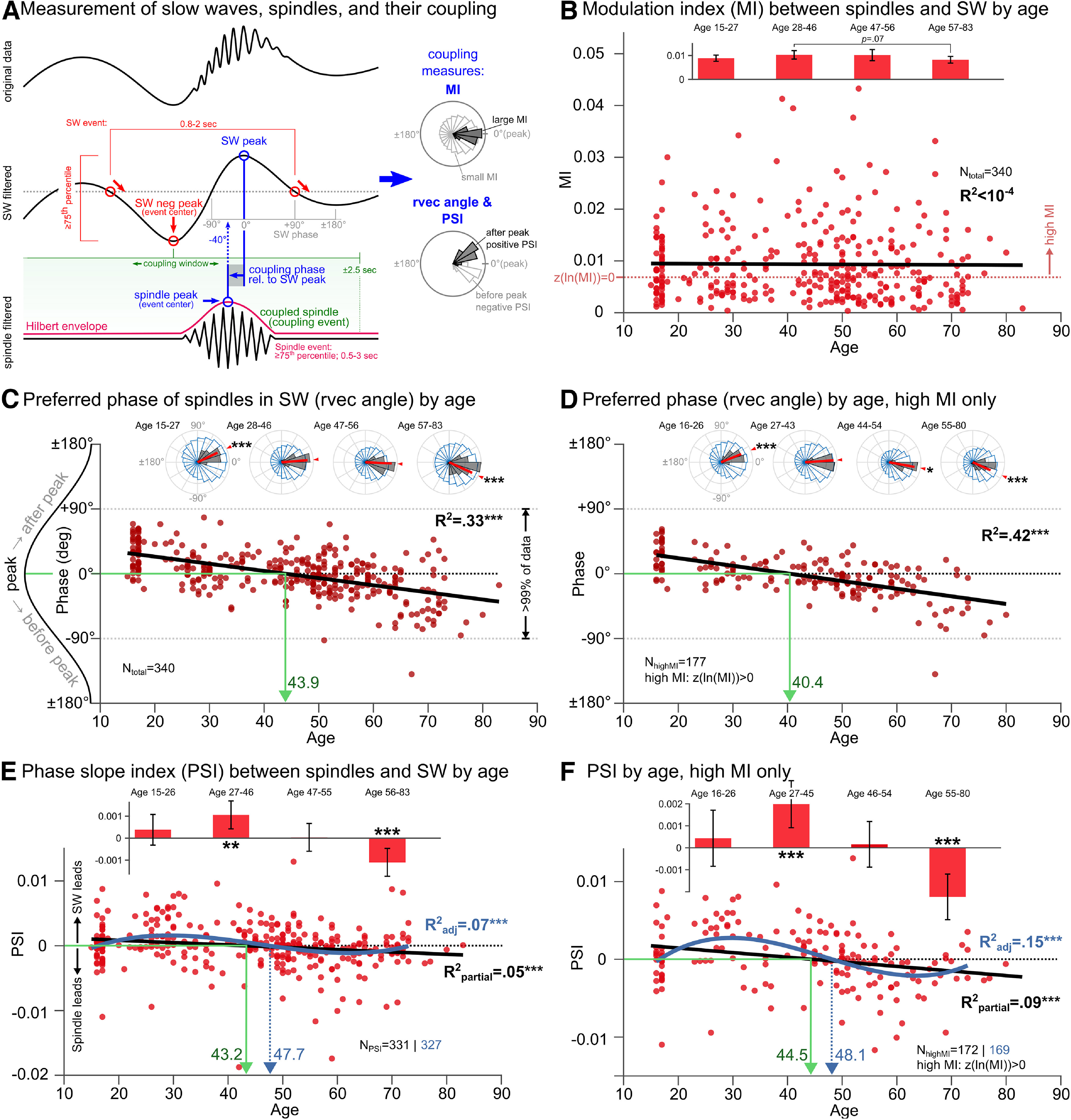
Slow-wave spindle phase–amplitude coupling across the human life span. ***A***, Illustration of measurement of SWs, spindles, and their coupling. In brief, SWs and spindles are detected using established duration and relative amplitude criteria (red and crimson). SW events are centered on their negative peak, and each spindle event is classified as SW coupled if it lies within 2.5 s of an SW event (green). Coupling (blue) is measured based on the SW phase occurring at the spindle peak (rvec angle; the average circular direction of spindle–SW coupling events), coupling strength (MI), and cross-frequency directionality (PSI; the consistency of phase lag or lead between two signals). A PSI significantly different from 0 suggests the leading signal drives the lagging signal. A positive PSI indicates SW drive spindles, and vice versa for negative PSI. ***B***, MI is not associated with age across the entire sample (*N*_total_), indicating that coupling strength does not change with age. Consequently, the high-MI subgroup analyses in panels ***D*** and ***F*** are not biased by age. The red dotted line separates high-MI from low-MI subsets at *z*(ln(MI)) = 0. Inset bar graphs show the means of MI in age quartiles Q1 to Q4. There was no overall group difference among age quartiles (*p* = 0.27), but pairwise comparisons revealed a trend for Q2 > Q4 (*p* = 0.07). ***C***, Circular–linear correlation of rvec angle with age across the entire sample (*N*_total_ = 340). 0° represents the peak of the SW, and ±180° represents the valley. While >99% of preferred coupling phases across all ages lie within the positive half-wave of the SW (i.e., between −90° and + 90°), there is a strong correlation of age and preferred coupling phase (*R*^2^ = 0.33). For younger individuals, spindles couple after the peak of the SW, while for older individuals, spindles couple before the peak of the SW. Inset phase histograms show the distribution of preferred coupling phases (dark bars) and all coupling events (light bars with blue outline) in age quartiles Q1 to Q4. For the youngest quartile (Q1), the average preferred coupling phase (red indicator) occurs significantly after peak, while for the oldest quartile (Q4), the average preferred coupling phase occurs significantly before the peak (small red arrows and vectors). The best-fit model suggests a reversal of coupling from after to before the SW peak at 43.9 years of age (green arrow). ***D***, Same as ***C***, but only in individuals exhibiting strong phase preference as measured by the MI between spindles and SW (*N*_highMI_ = 177; High MI = *z*(ln(MI)) > 0). In this sample, the relationship of preferred coupling phase and age is even more pronounced (*R*^2^ = 0.42). The best-fit model suggests a reversal at 40.4 years of age (green arrow), and Q3 already exhibits a significant shift of average preferred coupling phase to before the SW peak. ***E***, PSI between slow waves and spindles as a function of age. Data from nine participants were excluded because of unstable estimates (*N*_PSI_ = 331). A significant linear regression reveals a gradual reversal from SW leading spindles in younger individuals to spindles leading SW in older individuals (*R*^2^ = 0.03). When controlling for an age-related change in up-phase duration, this relationship becomes more pronounced (*R*^2^_partial_ = 0.05). The best-fit model suggests a reversal at 43.2 years of age. The inset bar graph shows age quartile means, *t*-tested against 0. Notably, the youngest quartile (Q1) does not show clear cross-frequency directionality, but Q2 and the oldest quartile (Q4) do, in line with the findings in ***C*** and ***D***. A stepwise linear model fitting higher-order polynomials resulted in a best fit using a cubic relationship (*R*^2^_adj_ = 0.07, blue curve), suggesting a rising and falling PSI across age (peaking at 29.2 years of age), potentially reflecting brain maturation processes in adolescents. ***F***, Same as ***C***, but only in individuals exhibiting strong phase preference as measured by the MI between spindles and SWs. In this sample, the relationship of PSI and age is more pronounced (linear, *R*^2^ = 0.07; linear, controlling for up-phase duration, *R*^2^_partial_ = 0.09; cubic, *R*^2^_adj_ = 0.14). The best-fit model suggests a reversal between 44.5 years of age (linear, green arrow) and 48.1 years of age (cubic, blue arrow). Note: for cubic relationships in ***E*** and ***F***, data above 73 years of age were excluded to prevent overfitting. *p < 0.05, **p < 0.01, ***p < 0.001.

Spindles prefer to nest into the positive half-wave of the SW for almost all participants across all ages, as 338 of 340 (>99%) of the individual rvec angles lay within SW phase angles of −90° and +90°. However, with age, the average preferred coupling angle shifts from after to before the peak of the SW. This is indicated by a significant circular–linear correlation of rvec angle and age (*r* = 0.57, *p* < 0.001), with age explaining 33% of the variance in rvec angle. For the youngest age-quartile (Q1), the average preferred coupling phase occurs significantly after peak (*M* = 24.8°, CI_95_ = [15.9°, 33.7°], *p* < 0.001), while for the oldest age-quartile (Q4) the average preferred coupling phase occurs significantly before the peak (mean = −22.0°; CI_95_ = −35.7°, −8.3°; *p* < 0.01). Age quartiles Q2 and Q3 did not exhibit significant deviations of rvec angle from 0°. The best-fit model suggests a reversal of preferred spindle coupling from after to before the SW peak at 43.9 years of age (Fig. 1*C*).

The age-dependent forward shifting of the preferred spindle-coupling phase within an SW becomes even more pronounced if participants exhibiting weak coupling are excluded. We repeated the circular–linear correlation of rvec angle and age only in participants exhibiting a high MI between SW and spindle frequencies. High MI was defined as *z*(ln(MI)) > 0 (Fig. 1*B*, data above the red dotted line), yielding a subgroup of *N*_highMI_ = 177. The resulting correlation was highly significant (*r* = 0.65, *p* < 0.001) with age explaining 42% of the variance in rvec angle, which is significantly higher than using the entire sample (Pearson–Filon *z*-test: −12.58, *p* < 0.001). In this high MI subgroup, age quartiles Q1 (mean = 20.6°; CI_95_ = 7.9°, 33.3°; *p* < 0.001), Q3 (mean = −6.9°; CI_95_ = −12.2°, −1.6°; *p* < 0.05), and Q4 (mean = −24.3°; CI_95_ = −43.3°, −5.2°; *p* < 0.001) show significant deviations of preferred coupling from the peak of the SW (0°), with a best-fit model-suggested reversal at 40.4 years of age (Fig. 1*D*).

The observed forward-shift of the preferred coupling phase with age manifested in a reversal of cross-frequency directionality. While in younger adults, SWs drive spindles, and in older adults spindles drive SWs. This is illustrated by a significant correlation of PSI and age (*r* = –0.19, *p* < 0.001). However, this measure allowing stronger claims exhibits higher variance compared with the preferred coupling phase using rvec angles, with age explaining only 3% of the variance in PSI. Notably, there was a shift in SW peak frequency across age, manifesting in a slightly increased duration of the up-phase (*R*^2^ = 0.01, *p* = 0.012), and a markedly increased duration of the down-phase (*R*^2^ = 0.26, *p* < 0.001) of SW events in line with previous reports (Carrier et al., 2011). Up-phase (*r* = 0.29, *p* < 0.001), but not down-phase (*r* = –0.05, *p* = 0.408) duration was correlated with PSI. PSI is inherently capable of addressing shifting frequency peaks (Jiang et al., 2015), especially since we chose a wider window for lower frequency between 0.5 and 2.0 Hz to allow for individual drifts. Still, this age-related shift in SW frequency may confound calculation of age-related trends in coupling. To account for this, we ran a stepwise optimized regression analysis initially including both the up-phase and down-phase durations of SW events as regressors. During optimization, down-phase duration was removed as a regressor, yielding a final model that revealed an improved effect of age on PSI (*F*_(2,328)_ = 21.7, *R*^2^_adj_ = 0.11, *p* < 0.001, age *R*^2^_partial_ = 0.05), indicating that varying SW frequency with age partially masked the effect of age on PSI.

Some studies suggest gender to be an interacting factor in age-associated changes in SWS (Ohayon et al., 2004; Redline et al., 2004) and, therefore, that gender could influence age-associated changes in SW–spindle coupling. Our stepwise regression optimization procedures were allowed to control for gender, but never included gender as a factor because it did not explain enough variance to pass the entry threshold. Still, we wanted to explicitly test for effects of gender using gender as an a priori regressor in a model explaining PSI by age and optimized this model stepwise using a more liberal threshold to keep terms (see Materials and Methods, subsection Testing for association of slow wave-spindle coupling and age). This model still showed a significant effect of age on PSI (*F*_(2,328)_ = 6.3, *R*^2^_adj_ = 0.031, *p* = 0.002, age *R*^2^_partial_ = 0.032), with gender having almost no influence (*p* = 0.62). Stepwise optimization reverted to the model above, removing gender but including SW up-phase duration.

Similarly, the COVID-19 pandemic may have influenced the sleep quality of participants enrolled in years 2020 and 2021. We repeated the procedure described above for gender, substituting gender for a regressor coding whether study participation occurred during the pandemic (true for 24 participants). This analysis yielded similar results, showing no effect of pandemic (*p* = 0.83) on the significant age-related change in PSI (*F*_(2,328)_ = 6.2, *R*^2^_adj_ = 0.031, *p* = 0.002, age *R*^2^_partial_ = 0.029). Again, stepwise optimization reverted to the model only including age and SW up-phase duration.

Importantly, age quartiles Q2 (mean = 0.0011; CI_95_ = 0.0004, 0.0017; *t*_(87)_ = 3.29; *p* = 0.001) and Q4 (mean = −0.0011; CI_95_ = −0.0017, −0.0005; *t*_(88)_ = −3.44; *p* < 0.001) exhibit significant deviations of PSI from 0, including a sign flip, indicating a reversal of which frequency drives the other at the best-fit model-suggested 43.2 years of age (Fig. 1*E*).

Interestingly, the youngest age quartile did not exhibit a significant PSI, indicating no clear cross-frequency directionality in the age group 15–26 years. To investigate a potential rising and falling relationship between age and PSI, a stepwise linear model was allowed to fit higher-order polynomial age terms. To prevent overfitting in data-sparse regions, data from four participants who were >73 years of age were removed from this model. Including a cubic polynomial peaking at 29.2 years of age resulted in the best model fit, increasing explained variance [change in Akaike information criterion (ΔAIC) vs linear: −11.95, *F*_(3,323)_ = 9.32, *R*^2^_adj_ = 0.07, *p* < 0.001). This model suggests a reversal of which frequency drives the other at 47.7 years of age (Fig. 1*E*, blue curve).

As with rvec angle, the age-dependent shift of PSI, including a sign flip, becomes more pronounced if only participants exhibiting strong coupling (high MI) are included (Fig. 1*F*). As with rvec angle, high MI was defined as *z*(ln(MI)) > 0, yielding a subgroup of *N*_highMI_ = 172. In this subgroup, the association of age and PSI became stronger, about doubling the explained variance. A linear correlation yielded *R*^2^ = 0.07 (*p* < 0.001), which is significantly stronger than including the entire sample (Pearson–Filon *z*-test: 33.14, *p* < 0.001). This association was again improved by including SW up-phase duration as a predictor in a linear regression (*F*_(2,169)_ = 19.9, *R*^2^_adj_ = 0.18, *p* < 0.001, age *R*^2^_partial_ = 0.09), and showed an even stronger cubic relationship (ΔAIC vs linear: −13.93, *F*_(3,168)_ = 10.50, *R*^2^_adj_ = 0.15, *p* < 0.001). A suggested reversal of which frequency drives the other was between 44.5 (linear) and 48.1 (cubic) years of age. This was again paralleled by age quartiles Q2 (mean = 0.0019; CI_95_ = 0.0009, 0.0030; *t*_(44)_ = 3.74, *p* < 0.001) and Q4 (mean = −0.0022; CI_95_ = −0.0033, −0.0012; *t*_(42)_ = −4.35, *p* < 0.001) exhibiting significant and opposite deviations of PSI from 0.

While SW–spindle coupling clearly shifts across the human life span, reversing the coupling hierarchy at ∼40–48 years of age, coupling strength remains unaffected. This is illustrated by the absence of an association of MI and age (*R*^2^ < 10^−4^, *p* = 0.84; Fig. 1*B*). An ANOVA on MI age quartiles did not yield a significant effect (*F*_(2,3336)_ = 1.32, *p* = 0.267). Pairwise comparisons showed a trend of Q2 > Q4 (*t*_(84)_ = 1.82, *p* = 0.070), which is reminiscent of previous findings (Helfrich et al., 2018), but this result is not robust and should be treated as a negative finding. In addition, the age groups where the effect seems to occur are not directly comparable (in the study by Helfrich et al., 2018, the mean ± SD age of the younger group was 20.4 ± 2.0 years, while the mean age of our Q2 group was 38.2 ± 6.3 years). Alternatively, the right-skewed nature of MI, combined with higher variance in the middle age quartiles compared with Q1/Q4 (Bartlett's χ^2^ = 34.93, *p* < 0.001) may cause the appearance of changing means.

### The lower the slow-wave frequency, the stronger the slow wave–spindle coupling and reversal of information flow

**Figure 2.**
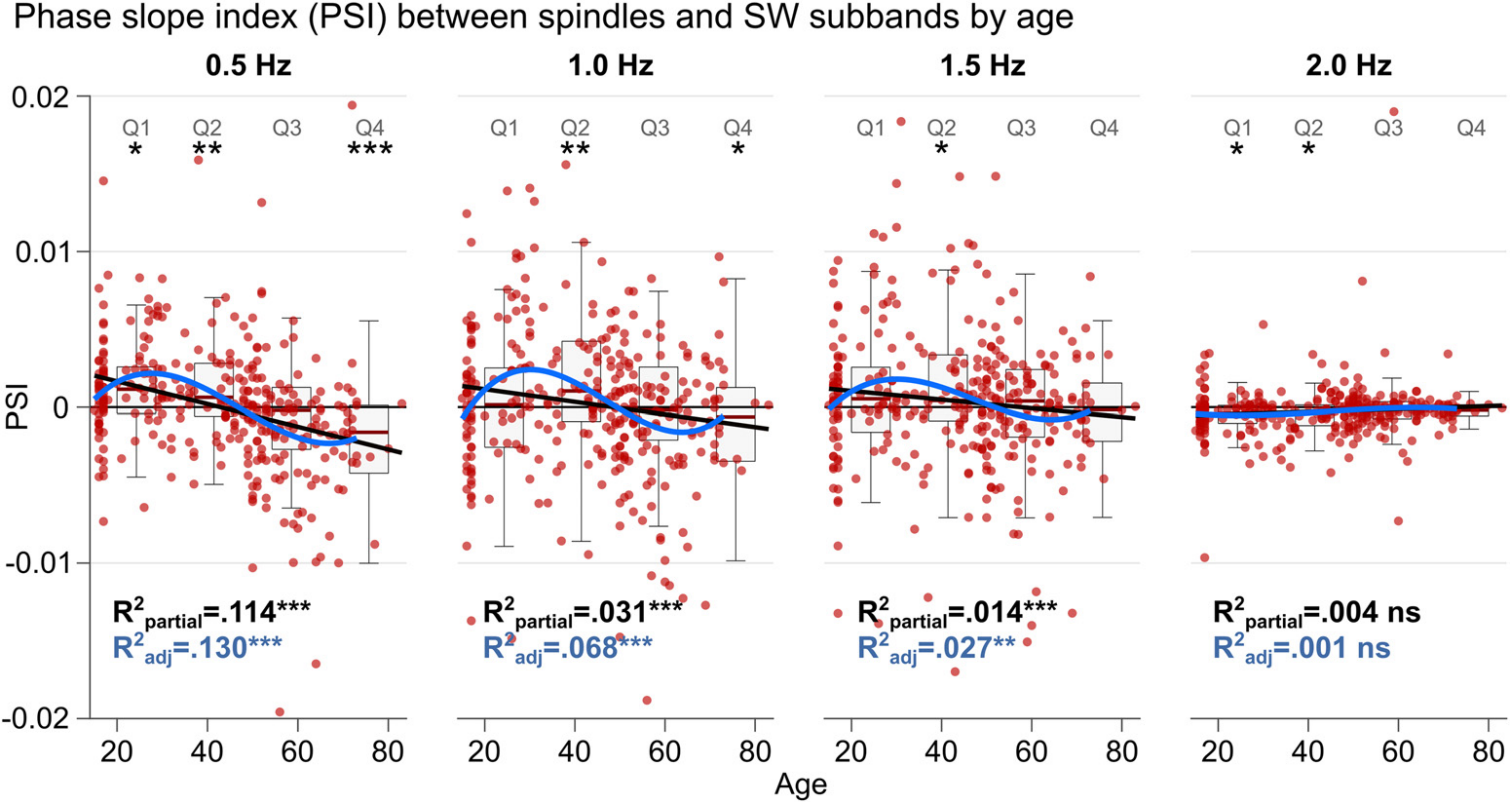
Cross-frequency directionality as measured using PSI between SW subbands (0.5, 1.0, 1.5, 2.0 Hz) and spindles (12–16 Hz) as a function of age. Data from nine participants were excluded because of unstable PSI estimates (*N* = 331). Coupling between SWs and spindles is strongest for the lowest SW frequency subband and gradually diminishes with increasing subband frequency, until it no longer is present for 2.0 Hz, marking the transition away from SW into δ frequency. Significant linear regressions, controlling for SW up-phase duration, show the reversal from SW leading spindles in younger individuals to spindles leading SW in older individuals for subbands 0.5, 1.0, and 1.5 Hz, but not 2.0 Hz (black lines). Cubic relationships follow the same trend (blue curves), suggesting a rising and falling PSI between SWs and spindles across age for SW frequencies ≤1.5 Hz. The lower the SW subband, the stronger the association. There is no linear or cubic association between age and PSI for the highest subband (2.0 Hz), which marks the transition away from SW into the δ frequency range. Boxplots show age quartiles (Q1 to Q4), *t*-tested against 0 (FDR corrected). *p < 0.05, **p < 0.01, ***p < 0.001, ^ns^*p* > 0.1.

To more closely investigate the exact nature of SW spindle coupling and the observed reversal of information flow (from SWs leading spindles to vice versa) with age, we reran the regression analyses of age on PSI for SW subbands (0.5, 1.0, 1.5, and 2.0 Hz) separately. A 4 × 4 repeated-measures ANOVA with the factors “SW subband” and “age quartile” (Q1 to Q4) revealed a significant main effect for SW subband (*F*_(2,3981)_ = 3.32, *p* = 0.033, Greenhouse–Geisser corrected) and a significant interaction with age quartile (*F*_(3,3981)_ = 7.37, *p* < 0.001). The significant interaction is because of lower SW subbands showing a stronger age-dependent reversal effect than higher subbands. Additional false discovery rate (FDR)-corrected *t* tests of single subbands against 0 indicated that significant sign flips from positive to negative PSI (i.e., reversal of information flow) occurred for subbands 0.5 and 1.0 Hz only (Fig. 2, compare asterisks for age quartiles).

Next, we investigated age trends for the four PSI SW subbands. For each SW subband, two models were calculated, paralleling the final models from the analysis of age on PSI in subsection Spindles shift from lagging to leading slow waves without loss of coupling strength with age and Figure 1*E*: (1) a linear model including SW up-phase duration as covariate; and (2) a model including up to cubic terms of age. The strongest age-related PSI shift occurred for the lowest SW subband, 0.5 Hz (linear: *F*_(2,328)_ = 28.4, *R*^2^_adj_ = 0.14, *p* < 0.001, age *R*^2^_partial_ = 0.11; cubic: *F*_(3,323)_ = 17.2, *R*^2^_adj_ = 0.13, *p* < 0.001; Fig. 2, left-most panel). With increasing SW subband frequency, this relationship got progressively less pronounced (1.0 Hz linear: *F*_(2,328)_ = 24.0, *R*^2^_adj_ = 0.12, *p* < 0.001, age *R*^2^_partial_ = 0.03; 1.0 Hz cubic: *F*_(3,323)_ = 9.0, *R*^2^_adj_ = 0.07, *p* < 0.001; 1.5 Hz linear: *F*_(2,328)_ = 9.1, *R*^2^_adj_ = 0.05, *p* < 0.001, age *R*^2^_partial_ = 0.01; 1.5 Hz cubic: *F*_(3,323)_ = 4.0, *R*^2^_adj_ = 0.03, *p* = 0.008; Fig. 2, middle panels). The highest SW subband, at 2.0 Hz, was no longer significantly associated with age (linear: *F*_(2,328)_ = 2.0, *R*^2^_adj_ = 0.01, *p* = 0.132, age *R*^2^_partial_ = 0.004; cubic: *F*_(3,323)_ = 1.2, *R*^2^_adj_ = 0.001, *p* = 0.31; Fig. 2, right-most panel). PSI exhibits markedly reduced variance in the highest SW subband (2.0 Hz), hovering at ∼0 (Fig. 2, right-most panel). This illustrates the transition away from slow-wave frequencies into the upper δ range. Counterintuitively, the first two age quartiles in the 2.0 Hz subband even exhibit significantly negative PSI, but because of the very low absolute values and variance, we treat this as a false-positive finding.

### Plasma GFAP, but not plasma amyloid β42/40, is associated with slow wave–spindle coupling in older individuals

**Figure 3.**
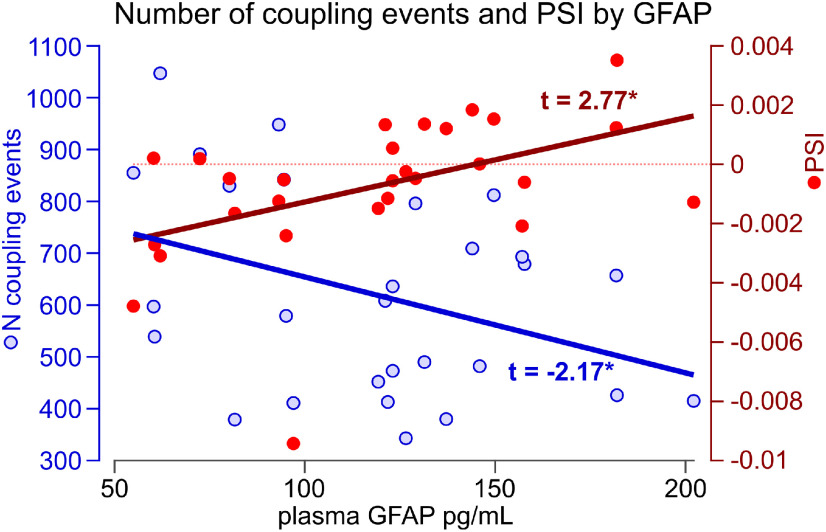
Association of number of SW–spindle coupling events (*N* coupling events, blue) and PSI (red) with plasma GFAP levels in older subgroup with biomarker measurements (*N* = 28; age range, 61–80 years). *N* coupling events and PSI significantly predict GFAP levels in an optimized linear regression (model: *F*_(2,25)_ = 6.45, *R*^2^_adj_ = 0.29, *p* = 0.006; see *t*-values for regressors in plot). No other terms were included during stepwise model optimization (i.e., age, gender, sleep parameters, and MI do not contribute to explaining GFAP levels). *p < 0.05.

Plasma GFAP levels after waking were strongly associated with SW–spindle coupling in 28 older individuals with biomarker measurements in an optimized linear regression model (*F*_(2,25)_ = 6.45, *R*^2^_adj_ = 0.29, *p* = 0.006). The number of coupling events (*t* = −2.17, *p* = 0.039) and PSI (*t* = 2.77, *p* = 0.010) were significant predictors of GFAP levels (Fig. 3). The negative association between the number of coupling events and GFAP levels indicates that individuals with lower overall SWS quality and/or quantity show increased signs of astrocyte activation. Somewhat counterintuitively, the positive association between PSI and GFAP indicates that older individuals exhibiting a more positive cross-frequency directionality typical for younger individuals (i.e., SWs driving spindles rather than spindles driving SWs) showed increased signs of astrocyte activation. Age and MI were dropped from the a priori baseline model, and no other terms (e.g., sleep parameters) were added during stepwise model optimization. Notably, as the proportion of neither N2/N3 sleep nor TST was considered a predictor of plasma GFAP levels, the association of the number of coupling events with GFAP seems to be independent of the absolute available time (quantity) in SWS for coupling to occur, and was more dependent on the quality of SWS determining whether coupling occurs or not.

The number of coupling events was best explained using an optimized model (*F*_(5,22)_ = 5.42, *R*^2^_adj_ = 0.45, *p* = 0.002) including all a priori terms (MI, PSI, age, and GFAP), as well as TST as a strong predictor. While MI (*t* = 2.20, *p* = 0.039) and TST (*t* = 3.09, *p* = 0.005) significantly predicted the number of coupling events, PSI (*t* = 1.63, *p* = 0.118), age (*t* = −1.74, *p* = 0.096), and GFAP levels (*t* = −1.69, *p* = 0.106) explained enough variance to remain in the model. Unsurprisingly, the number of coupling events increases with TST, illustrating its dependence on sleep quantity. The positive association of MI and coupling events, on the other hand, explains how qualitative aspects of SWS, as indicated by coupling strength, are associated with an increased occurrence of coupling events.

MI was best explained using an optimized model (*F*_(4,23)_ = 4.00, *R*^2^_adj_ = 0.31, *p* = 0.013) including the number of coupling events (*t* = 2.27, *p* = 0.033), PSI (*t* = −2.68, *p* = 0.013), GFAP (*t* = 0.48, *p* = 0.638), and the interaction of PSI * GFAP (*t* = 2.22, *p* = 0.036). Age was removed from the model during stepwise model optimization, paralleling the result of the whole-sample analysis (*N* = 340; Fig. 1*B*).

PSI was best explained using an optimized model (*F*_(3,23)_ = 3.62, *R*^2^_adj_ = 0.23, *p* = 0.028) including the number of coupling events (*t* = 1.36, *p* = 0.19), MI (*t* = −1.62, *p* = 0.118), and GFAP (*t* = 2.54, *p* = 0.018), excluding age. Although the number of coupling events and MI explained enough variance to stay in the model, GFAP was the only term significantly explaining the variance in PSI.

Plasma Aβ42/40 ratios were not explained by any of the available measures (*F*_(1,26)_ = 1.40, *R*^2^_adj_ = 0.01, *p* = 0.248). The number of coupling phases, PSI, and age were dropped from the a priori baseline model, and MI remained as a nonsignificant predictor in the optimized model (*p* = 0.248), indicating that amyloid clearance is seemingly not related to SW–spindle coupling in healthy older adults.

**Figure 4.**
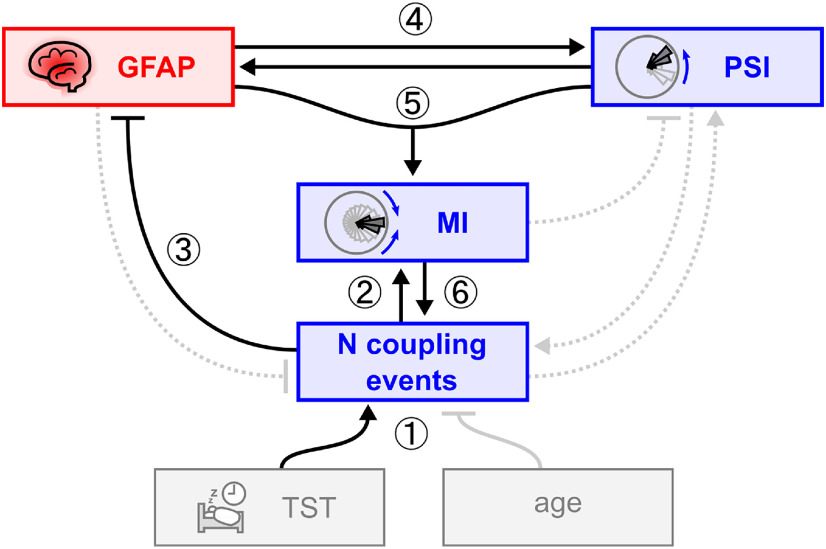
Regressor structure of optimized linear models in older subgroup with biomarker measurements (*N* = 28; age range, 61–80 years). Models were calculated for linear SW–spindle coupling measures (PSI, MI, number of SW–spindle coupling events; *N* coupling events; in blue) and plasma GFAP levels (a biomarker for astrocyte activation; in red). Additional variables are TST and age (in gray). Pointer lines indicate regressors for pointees. Arrows are positive associations, T-ends are negative associations. Black lines are significant regressors (*p* < 0.05), the solid gray line is a trend (*p* = 0.096), and gray dotted lines are nonsignificant regressors explaining enough variance to remain in models (i.e., the model *R*^2^ would drop by >0.05 if removed). The converging pointer from GFAP and PSI to MI indicates the significant interaction GFAP * PSI on MI. We hypothesize the following model to explain this regressor structure: ① With age, sleep becomes fragmented, reducing the available time for SW–spindle coupling to occur. The reduction in *N* coupling events is associated with ② a decrease in MI and ③ an increase in plasma GFAP, suggesting decreased coupling quality and increased astrocyte activation, potentially because of deteriorating neural integrity. ④ Notably, an increase of plasma GFAP is associated with an increase in PSI, suggesting an astrocyte activation-associated shift of coupling phase toward the physiology of a younger brain. ⑤ This shift, in interaction with the increase in GFAP, is in turn associated with an increase in coupling strength (MI), ⑥ which is positively associated with *N* coupling events. The positive association between PSI and GFAP ④ is unexpected and may be explained in the following two ways. (1) Coupling phase (PSI) is backshifted toward a younger state to compensate for the suboptimal development of sleep quality, coupling physiology and astrocyte activation. This backshift improves coupling strength directly and may indirectly lead to more overall coupling (*N* coupling events). (2) Alternatively, the forward-shifted coupling phase observed across the human life span (Fig. 1) is a normal physiological process, and a lack of this shift (as indexed by an age-relative positive PSI) is suboptimal and associated with astrocyte activation. We favor the compensatory explanation (explanation 1) because the age-associated forward shift in coupling phase has been shown to be associated with neurodegeneration (Helfrich et al., 2018), and an age-relative backshift has been associated with improved brain integrity and memory (Muehlroth et al., 2019).

In summary, these optimized models suggest a link between SW–spindle coupling and astrocyte activation: falling sleep quality-related and quantity-related reduction in SW–spindle coupling events was associated with increased signs of astrocyte activation as measured by plasma GFAP levels (Figs. 2, 3). Increased GFAP levels in turn were paralleled by a shift in PSI resembling the physiology of younger participants. Age is not directly associated with this process, suggesting this older age subgroup to be of homogeneous age. We deliberate on potential explanations (e.g., compensatory increase in PSI in response to deteriorating neurophysiology and neural integrity) in the Discussion section and Figure 4.

## Discussion

We show that SW–spindle phase–amplitude coupling gradually shifts across the human life span without losing coupling strength. Corroborating previous reports (Mikutta et al., 2019; Muehlroth et al., 2019; Winer et al., 2019), SWs drive spindles in younger individuals, representing the canonical hierarchy of top-down neocortical control of information flow (Rasch and Born, 2013; Staresina et al., 2015). However, while others report that this hierarchical structure dissipates with age (Helfrich et al., 2018), we found that the hierarchy reverses, settling into a configuration of spindles driving SWs in old age. The extent of this reversal of coupling hierarchy was associated with markers for astrocyte activation.

Importantly, we demonstrate a gradual, not sudden, forward-shift of SW–spindle coupling across the adult human life, paralleling another large-sample study (McConnell et al., 2021). This gradual nature notwithstanding, this shift results in a fundamental structural change—a reversal of the order of events and thereby the hierarchical structure observed in younger adults—starting at ∼40–48 years of age. In old age, spindles shift from being driven by SWs to driving SWs. This effect was stronger the lower the SW-subband that was analyzed: 0.5 Hz exhibited the strongest shift and coupling (PSI) with spindles, 1.0–1.5 Hz exhibited gradually reduced shift and coupling, and 2.0 Hz exhibited no shift/coupling, marking the transition away from SW and into the upper δ frequency band, which no longer seems to orchestrate sleep oscillations. What exactly the downstream effects of this shift regarding information flow inside the brain networks are must be speculated on, but others suggest that a precise hierarchy of SWs driving spindles is not a necessity for information processing, but helps to make it more efficient (Muehlroth et al., 2019). These authors find that in old age, a coupling hierarchy reminiscent of a younger brain is associated with higher structural integrity in key brain regions for memory processing (e.g., hippocampus and medial prefrontal cortex). This hints toward the existence of mechanisms for the preservation the physiology of a younger brain, or potentially for the compensation of loss thereof. This dovetails with findings that lifelong learning and cognitively stimulating environments contribute to cognitive fitness and neuronal integrity, aid the clearance of amyloid β, and may counteract cognitive decline (Brown et al., 2003; Lazarov et al., 2005; Fischer et al., 2007; Flexman, 2021).

Our subgroup analysis relating astrocyte activation, and therefore potential neuroinflammatory processes, to SW–spindle coupling provides a result that dovetails into such a model of maintenance and/or compensation. We find that age-related loss of sleep leads to reduced coupling. Reduced coupling is associated with an increase in plasma GFAP, a biomarker for astrocyte activation and a warning sign for potential impending cognitive decline and Alzheimer's disease. Interestingly, increased astrocyte activation is accompanied with a backshift of SW–spindle coupling toward the physiology of a younger brain. This backshift, in turn, is associated with an increase in coupling strength and indirectly may lead to more coupling overall. This could be an indication that the aging brain attempts to compensate for loss of sleep and structural integrity by shifting the coupling hierarchy back into a more optimal configuration. An alternative explanation would be that the age-associated reversal of coupling hierarchy is a normal, healthy process, and a failure to do so is a sign of a suboptimal development, paralleled by astrocyte activation. However, as other studies strongly indicate that the age-related forward-shift in SW–spindle coupling physiology is a detrimental development associated with memory loss and brain atrophy (Ladenbauer et al., 2017; Helfrich et al., 2018; Muehlroth et al., 2019; Chylinski et al., 2022), we find this alternative explanation to be unlikely.

A notable contrast to previous findings (Helfrich et al., 2018) is that here coupling strength (MI) did not change as a function of age. Our results indicate that MI does not exhibit age-related changes in the mean, but rather in variance, resembling an inverted U-shaped curve, with middle age quartiles exhibiting larger variance in MI than extreme age quartiles (Fig. 1*B*). This may lead to the following spurious changes in the mean: MI is right skewed and cannot become negative. The lower variance in Q1 and Q4 thus leads to an asymmetrical absence of high (but not low) MI values that causes a lowered mean. The analysis in the original report by Helfrich et al. (2018) may have captured this effect, but its larger dynamics across the life span remained hidden from those authors as they only had access to distinct age groups.

The change in coupling phase with concomitant stability of coupling strength might explain why declarative memory is generally more severely impacted in aging and neurodegeneration compared with procedural memory (Tromp et al., 2015), as declarative memory has been associated with coupling phase, while procedural memory has been associated with coupling strength (Mikutta et al., 2019).

We found that the typical coupling hierarchy (as measured using PSI) of younger adults does not yet exist in our youngest age quartile, although the overall event order typical for younger adults (spindles after SW peak, measured using rvec angle) is already established. This hints toward a dissociation between the mere order of events versus the leading event exerting influence over the lagging event. This quartile spanned ages 15–26 years, with adolescents under the age of 18 years featuring prominently. Only in the second quartile with ages ranging from 27 to 46 years, the canonical young adult coupling hierarchy (PSI > 0) is established. Our data-driven model suggested that a nonlinear relationship exists between coupling hierarchy and age, with an early “adolescent-young adult” and a later “adult life span” component. During the early component, the canonical hierarchy is established, peaking at age 29.2 years, and subsequently shifts gradually into the reported reversed hierarchy during the later component. This early component tracks with brain maturation, especially of white matter, which continues well into young adulthood of the early 20s (Konrad et al., 2013). Paralleling our finding, a recent study found that SW–spindle coupling strength increases during childhood into adolescence and is associated with enhanced memory formation (Hahn et al., 2020). We find a similar inverted U-shaped dynamic in changing variance in coupling strength (MI) with age. Taken together, the nonlinear waxing and waning of the SW–spindle coupling hierarchy and, arguably, coupling strength across the human life span may reflect different biological processes: maturation, plateau, and wear.

### Limitations

Our study has several limitations. Although we were able to investigate a large sample of baseline sleep recordings, we were limited to a single frontal EEG derivation, and there were no behavioral tasks available to associate memory, executive functions, or other cognitive domains to SW–spindle coupling. However, among other studies that did measure memory, there is a strong consensus that a “younger” SW–spindle coupling physiology is optimal for memory consolidation and that age-related changes in coupling are associated with reduced memory performance (Ladenbauer et al., 2017; Helfrich et al., 2018; Mikutta et al., 2019; Muehlroth et al., 2019; Bastian et al., 2022; Chylinski et al., 2022).

We could only assess the association of SW–spindle coupling with blood-based biomarkers in a subset of 28 older individuals. The lower statistical power of this comparatively small subset may explain why we were not able to find an association with plasma amyloid levels. However, this lack of association between coupling and amyloid is consistent with other studies (Winer et al., 2019, 2020), although one report finds that a forward-shift of spindles was associated with Aβ burden in the medial prefrontal cortex and memory decline (Chylinski et al., 2022). Arguably, aberrant amyloid dynamics, although predictive of cognitive decline years in advance (Beyer et al., 2022) may not be prominent enough in healthy older adults (yet) to associate with sleep microstructural dynamics like SW–spindle coupling (Winer et al., 2020).

Based on an ample body of literature, we speculate that increased GFAP levels may be indicative of neuroinflammation (Manchanda et al., 2018; Verberk et al., 2021; Beyer et al., 2022; Xiao et al., 2022). However, GFAP levels are also associated with general and benign astrocyte activation (Verkhratsky and Nedergaard, 2018). Finally, our stepwise regression method is rather exploratory in nature. The suggested mechanistic pathway model attempting to explain the association of astrocyte activation/neuroinflammation with a younger coupling physiology is hypothetical, with directions of causality not resolved. We interpreted the regressor structure in a way that made sense in the context of other studies. However, more research is needed to confirm or refute this model, including human intracranial recording studies for more direct physiological measurements or animal studies directly manipulating cellular processes and assessing biomarker responses (Katsuki et al., 2022).

### Conclusions and future directions

Our results generally agree with those of previous studies. However, the specific finding that SW–spindle coupling shifts across the human life span without losing coupling strength, with a reversal of the typical hierarchical coupling structure at midlife, is a novel finding in slight contrast with previous reports (Helfrich et al., 2018). It has generally been the assumption that the tight SW–spindle coupling typically seen in younger individuals becomes fuzzier in old age, but we do not see a decrease in coupling strength or a dissolution of a clear hierarchical structure of cross-frequency directionality in our data. On the contrary, we see that in the oldest age quartile, a hierarchical structure of cross-frequency directionality reemerges, but in reversed form, with spindles driving SWs. Zooming into this older age group, we find that deteriorating sleep, coupling physiology, and astrocyte activation go hand in hand. Astrocyte activation is associated with a hierarchical backshift of cross-frequency directionality to a younger status, potentially indicating compensation.

This assumption of compensation is exploratory and should be followed up with more systematic, prospective studies. However, if the model holds, it may hint toward SW–spindle coupling during sleep as a potential target for intervention against or prevention of cognitive decline. As the process needing to be reversed (i.e., the shifting coupling hierarchy) starts to gradually, not suddenly, shift into a qualitatively different configuration at midlife, and as the GFAP/amyloid biomarker profile can be used to predict neurodegeneration up to 17 years before onset (Beyer et al., 2022), there remains ample time to intervene. This potentially enables early, low-threshold, “soft” lifestyle adjustments to serve as a sufficient push in the right direction to avoid pathologic trajectories, saving resources and preserving quality of life for otherwise afflicted individuals. What these adjustments might be should be investigated further, but, as our model suggests a connection between total sleep time and coupling physiology, a focus on good sleep hygiene throughout one's life would be a good starting point.
